# A Multicriteria Approach for Measuring Employee Well-Being

**DOI:** 10.3389/fpsyg.2022.795960

**Published:** 2022-05-27

**Authors:** Junjie Dong, Shumin Yan

**Affiliations:** School of Economics and Management, Tongji University, Shanghai, China

**Keywords:** employee well-being, job satisfaction, life satisfaction, multicriteria approach, fuzzy measure

## Abstract

This paper proposes that employee well-being includes four dimensions: job satisfaction, life satisfaction, positive affect, and negative affect. Each dimension is interdependent and correlated. Therefore, the measurement of employee well-being is complicated and fuzzy. This study aims to treat the measurement of employee well-being as a fuzzy problem, construct a measurement model from the perspective of multi-criteria decision making, and establish the preference relationship between indicators through fuzzy measure and Choquet integral. Applying multiple linear regression analysis and the heuristic least mean squares method, the main findings are as follows: (1) It is inappropriate to use job satisfaction as a substitute for measuring employee well-being, as the weight of job satisfaction is the lowest among the four dimensions. (2) Employee well-being is also largely reflected in their overall satisfaction with life because life satisfaction is the most heavily weighted. (3) Employee well-being needs to consider the emotion-related indicators and satisfaction-related indicators comprehensively because fuzzy analysis proves that their relationship is redundant. Finally, the practical implications of these findings and future research directions are discussed.

## Introduction

Employee well-being is a necessary but not sufficient indicator of a good life and a good society. Although employees attach more importance to the openness and freedom of the organization as well as the meaning and pleasure of work itself, the practice of the company brings increasing work pressure, and vicious events such as sub-health, depression, karoshi, and suicide. The contradiction between the internal stressors in the workplace and employees’ pursuit of happiness makes employee well-being become one of the trends in organizational behavior research. Studies have shown that a high level of employee well-being is related to work engagement (Bakker et al., 2014), optimal job performance (Bakker and Oerlemans, 2011), and job satisfaction (Judge et al., 2001). Many Chinese companies, including Microsoft Research Asia, Suning, and Alibaba, have launched various initiatives and programs to improve employee well-being.

Academic researchers have a great interest in employee well-being, and thousands of literature have analyzed employee well-being from the idea of conceptual theory or empirical research. In recent years, employee well-being is not only emphasized in the field of psychology and organizational behavior, but also has been intersected with a variety of disciplines, including economics (Bencsik and Chuluun, 2021), sociology (Blanco-Encomienda et al., 2020), and computer science information system (Lawrance et al., 2021). However, the academic focus on employee well-being has not inspired enthusiasm for the measurement of this variable. A large number of existing literature generally use job satisfaction to measure employee well-being in the workplace, which is biased. Just as subjective well-being (SWB) includes overall life satisfaction, positive emotions, and negative emotions (Diener, 2009b), we believe that employee well-being also includes multiple components, such as job satisfaction, life satisfaction, positive emotions, and negative emotions. There are several reasons: Firstly, the definition and connotation of employee well-being are largely unresolved and ununified, there are also differences in terms of employee well-being in different literature, such as quality of work life (Alrawadieh et al., 2020), work-related quality of life (McFadden et al., 2021), workplace well-being (Duan et al., 2019), employee mental health (Han and Hyun, 2019), etc. Therefore, the concept of employee well-being can be said to be dynamic and multidimensional. Secondly, job satisfaction includes the degree of work satisfaction and the feelings related to the work (Zheng et al., 2015). It has been criticized as the improper operation of happiness at work to measure the overall well-being at work by job satisfaction (Wright and Cropanzano, 2004). Wright and Bonett (2007) reveal that job satisfaction is an effective predictor of performance, while employee well-being moderates this effect. Therefore, it is fragmentary to measure employee well-being only by job satisfaction. Thirdly, job satisfaction, life satisfaction, positive emotions, and negative emotions are all correlated. An integrated model indicates that job satisfaction is significantly related to life satisfaction (Rode, 2004), positive and negative emotions vary over time and help predict participants’ job satisfaction that day (Ilies et al., 2006).

For these three reasons mentioned above, it is reasonable to believe that the measurement of employee well-being is complex and vague, and cannot be precisely defined or expressed with a clear value. In this case, we treat the measurement of employee well-being as a fuzzy problem and introduce the idea of multi-criteria decision-making (MCDM). In general, MCDM is a methodological and modeling tool for complex engineering problems, in which decision-makers are faced with problems of incomplete and vague information. Fuzzy set theory is applicable when human knowledge needs to be modeled and human assessment needs to be performed, it allows for solving a lot of problems related to dealing the imprecise and uncertain data (Balmat et al., 2011) and has been successfully applied and implemented in MCDM for many years. Since Murofushi (1992) proposed the idea of using Shapley value as an important index, and later his introduction of the interaction index (Murofushi and Soneda, 1993), fuzzy measures, as well as the Choquet integral, have been efficiently used for dealing with MCDM problems. So far, the application of MCDM has been extended to other fields besides engineering, for instance, Lang et al. (2021) propose a multi-criteria approach to plan attended home deliveries profitably and efficiently, Pereira et al. (2020) develop a multi-criteria decision approach to rank European health systems, Clemente-Suárez et al. (2021) highlight the importance of fuzzy multi-criteria decision analysis for emergency response systems related to the COVID-19 pandemic.

Based on the existing literature, this study proposes that the connotation of employee well-being includes multiple dimensions of job satisfaction, life satisfaction, positive emotion, and negative emotion, and each dimension is related to the other. Therefore, we regard the measurement of employee well-being as complicated and fuzzy. The purpose of this study is to reconstruct employee well-being using MCDM and to model the preference relationship between employee well-being criteria through fuzzy measure and Choquet integral. This paper makes contributions in the following ways: (a) This study enriches the existing research on employee well-being, which is a supplement to existing literature. (b) This study introduces the idea of MCDM and fuzzy measure, which is a kind of interdisciplinary intersection and helps to interpret employee well-being from a different perspective. (c) Based on the research conclusions, this study puts forward relevant suggestions on how to improve employee well-being in the workplace, which has important practical significance for the development of the organization.

## Literature Review

Employee well-being has been widely concerned and studied in the field of organizational behavior, but there is no unified definition and connotation. International Labor Organization (ILO) claims that employee well-being in the workplace involves all aspects of their working life, from the safety and quality of the physical environment to the employees’ feelings about work, work environment, organization, and work atmosphere (Ip, 2009). It can be seen that employee well-being involves all aspects of employee perception, but most empirical studies use job satisfaction to measure employee well-being (Inceoglu et al., 2018), which is biased. Existing research generally agree that SWB has three core components: a high level of positive emotion, a low level of negative emotion, and cognitive evaluation of overall life satisfaction (Diener et al., 1999; Page and Vella-Brodrick, 2009). According, we believe there can be four main components of employee well-being: the presence of positive affect, the absence of negative affect, life satisfaction, and job satisfaction.

### Positive and Negative Affect

Positive and negative affect reveal basic experiences of what is going on in people’s lives, there is no doubt that many agree that these emotional assessments should be the basis for SWB judgments (Kahneman, 1999). Positive affect is closely associated with hedonism, making people feel alert and engaged, while negative affect is associated with psychophysiological pain (Salavera et al., 2020). Research in SWB usually focus on emotional dimensions rather than specific emotions because different emotions with the same valence are moderate to strongly correlated over long periods (Zelenski and Larsen, 2000). For instance, people who experience a high level of depression may also experience other negative emotions, such as anxiety or fear. Because these emotions are interrelated, they may come from the same underlying processes, and their fundamental dimensions are the basis for the covariation of emotions that people experience.

Most emotional models focus on two basic dimensions. For example, Russell (1980) believed that the orthogonal dimensions of arousal and pleasantness can be used to explain the changes in emotional experience. Further, according to Watson and Tellegen (1985), the arousal and pleasantness dimensions should be rotated by 45° to form a separate dimension that activates positive and negative influences. The controversies about the structure of emotional models have led to a debate about whether positive and negative emotions are independent and separable dimensions. Diener and Emmons (1984) suggested that positive and negative affect were biphasic at any given moment, but they became independent as they cluster together over time. According to this view, individuals cannot experience both positive and negative affect at the same time, but they can experience high levels of both over time. However, Larsen et al. (2001) believed that positive and negative affect can be experienced at the same time under unusual circumstances. Whatever the conclusion of this debate, it seems more reasonable to assess the positive and negative affect separately, especially given the correlation between the two.

There are three major scales for measuring shorter-term emotions. The most outstanding one is the Positive and Negative Affect Schedule (PANAS) developed by Watson et al. (1988), it is a self-report scale about how respondents feel during the past few days or weeks, and its validity has been widely validated based on classical test theory (Medvedev et al., 2021). The second one is the Swedish Core Affect Scale (SCAS) proposed by Västfjäll et al. (2002), it measures core affects, which are considered as an implement for understanding emotions and vary along two orthogonal dimensions defined as activation and valence. A recent alternative is the Scale of Positive and Negative Experience (SPANE) introduced by Diener et al. (2010), it generates a score for positive affect (6 items), a score for negative affect (6 items), and finally creates a balance score.

### Life Satisfaction

Life satisfaction is a type of assessment with global judgments about one’s life. Individuals can evaluate the situations in their lives, assign weight to those situations, and then evaluate the quality of their lives ranging from satisfied to dissatisfied (Diener et al., 2009a). Existing literature suggests that life satisfaction is influenced by many factors, for example, work characteristics such as work-life balance and job insecurity (Viñas-Bardolet et al., 2020), religious services (Speed and Lamont, 2021), and fear of robots (Hinks, 2021). However, individuals’ judgments of life satisfaction are based on instant information at the time of their judgments, and most of this information remains constant over time, thus it is reasonable to believe that individuals’ life satisfaction judgments have a considerable degree of temporal stability (Ehrhardt et al., 2000). Also, the weight of this information varies from person and culture. For example, Suh et al. (1998) found that individualism is more dependent on emotional well-being than collectivism when judging life satisfaction. In terms of individual differences, daily happiness experience has a greater predictive effect on life satisfaction of individuals with high sensation-seeking than those with low sensation seeking (Oishi et al., 2001).

Generally, global life satisfaction is measured by the Satisfaction with Life Scale (SWLS) developed by Diener et al. (1985). The items in the SWLS are global rather than detailed in nature, allowing participants to measure various areas of their lives according to their values, thus leading to a global judgment of life satisfaction. This scale asks respondents to what extent they agreed with the five descriptions, with responses ranging from “strongly disagree” to “strongly agree” on a 7-point scale. However, the SWLS does not assess all aspects of SWB, it aims to measure the cognitive component of SWB rather than the emotional component, thus SWLS scores cannot automatically be used as a direct measure of emotional well-being (Diener, 2009a).

### Job Satisfaction

In the research of organizational behavior, the feature of employee well-being can be outlined as job satisfaction, because job satisfaction “implicitly captures well-being relative to outside job opportunities” (Green, 2010). Locke (1969) defined job satisfaction as an enjoyable emotional state caused by the evaluation of one’s job. Employees who are satisfied with their jobs may experience a high degree of pleasure but may have limited aspirations and energy (Grebner et al., 2005). Studies have shown that job satisfaction is affected by the following factors: job crafting strategies (Li et al., 2021), social support (Chen et al., 2021), and personal development opportunities (Heimerl et al., 2020). But it is still uncertain whether job satisfaction is a suitable predictor of employee well-being (Bryson et al., 2017) because others have pointed out that the measurement of job satisfaction ignores the emotional component and focuses on the cognitive part (Brief and Weiss, 2002; Weiss, 2002). For this reason, three widely used scales require descriptions and assessments of specific job features, rather than just evaluations of job feelings or job satisfaction: the Minnesota Satisfaction Questionnaire (MSQ) by Weiss et al. (1967), the Job Descriptive Index (JDI) by Kendall and Hulin (1969), and the Job in General Scale by Ironson et al. (1989).

In addition to the above scales, researchers have employed a multiple-measure approach to measuring workplace well-being in recent years. For example, Cotton and Hart (2003) assumed the measurement of employee well-being contained both positive and negative affect along with the cognitive assessment of job satisfaction. Page and Vella-Brodrick (2009) suggested that employee well-being should include both job-related psychological experience and non-job-related psychological experience and health status. Ilies et al. (2007) conceptually classified employee well-being into individual and situational factors and further divided it into job-related and non-job-related factors. In line with this trend, Vanhala and Tuomi (2006) proposed that the measurement of employee well-being should not only consider employees’ work and health but also assess their family relationships and life satisfaction. Zheng et al. (2015) developed a multidimensional scale to measure employee well-being consisting of life well-being, workplace well-being, and psychological well-being.

While each variable has its definition and measurement, it does not imply that they are independent. For example, satisfaction with life as a whole may spill over to job satisfaction, and some literature suggest that job satisfaction is one of the determinants of life satisfaction (Rode, 2004; Bialowolski and Weziak-Bialowolska, 2021). The correlation analysis of Jovanović and Joshanloo (2021) indicates that life satisfaction is significantly positively correlated with positive emotions and negatively correlated with negative emotions. Moreover, studying only the positive or negative emotions of employees is not meaningful because employees may experience a wide variety of emotions at work, each of which may be important in predicting valuable organizational outcomes (Page and Vella-Brodrick, 2009). Although each component of SWB has a unique positive or negative impact on employees, these effects are interdependent and interactional. Combining the measurements of these concepts with each other can account for additional redundancy and variance. Therefore, we believe that the measurement of employee well-being is a fuzzy problem, which can be solved by using the idea of MCDM.

## Materials and Methods

### Fuzzy Measure and Fuzzy Integral

The principal characteristic of fuzzy measure is that it can express the interaction relationship among criteria, this is because each subset of criteria is given a weight of importance.

For the convenience of introducing fuzzy measure and fuzzy integral here, let *N* = (1,2,…,*n*), *n*≥2 be a decision criterion set and 𝒫(*N*) be the power set of *N*.

Definition 1. A fuzzy measure (or called capacity, non-additive measure) on *N* is a monotonicity set function μ:𝒫(*N*)→[0,1] satisfying

*(i)* μ(ϕ) = 0, μ(*N*) = 1;

*(ii) for* ∀*A*⊆*B*⊆*N* implies μ(*A*)≤μ(*B*) (monotonicity).

Murofushi and Sugeno (1989) put forward an abstract interpretation of fuzzy measure: the non-additivity of fuzzy measures representing the interaction between subsets. According to this interpretation, there can be two types of interactions between two disjoint subsets *A*, *B*:

• Redundant or negative synergy: μ(*A*∪*B*) < μ(*A*) + μ(*B*), which indicates the importance of the union of these two disjoint subsets is no better than the importance sum of these two individual disjoint subsets.
• Complementary or positive synergy: μ(*A*∪*B*) > μ(*A*) + μ(*B*), which indicates the importance of the union of these two disjoint subsets is better than the importance sum of these two individual disjoint subsets.

Fuzzy integral is an integration function concerning fuzzy measures. The most typical fuzzy integrals are the Sugeno integral and Choquet integral. In this paper, we mainly employ Choquet integral for integration, which can be used in any positive function. The principal advantage of the Choquet integral is that it coincides with the Lebesgue integral when the measure is additive.

Definition 2. Let μ be a fuzzy measure on *N*, the Choquet integral of a function *f*:*N*→ℝ^+^ concerning μ is defined by


(1) $$C_{\mu }\,\left(f\right):=\sum_{i=1}^{n}f_{\sigma \,\left(i\right)}\,\left[\mu \,\left(A_{\sigma \,\left(i\right)}\right)-\mu \,\left(A_{\sigma \,\left(i+1\right)}\right)\right]$$

where σ is a permutation on *N* so that *f*_σ(1)_≤*f*_σ(2)_≤…≤*f*_σ(*n*)_. *Also*, *A*_σ(*i*)_:=(σ(*i*),…,σ(*n*)), for all *i* ∈ (1,…,*n*), and *A*_σ(*n*+1):=∅_.

According to the definition of fuzzy measure, we note that the importance of criterion *i* depends on the values of μ(*i*,*K*), where *K* ∈ *N*. Shapley (1953) proposed an importance index called Shapley value on an axiomatic basis in cooperative game theory, which possesses all appropriate properties for representing the importance of every criterion.

Definition 3. Let μ be a capacity on *N*, the Shapley value of a criterion *i* ∈ *N* is defined as


(2) $$I_{i}=\sum_{A\subseteq N\,\left(i\right)}\frac{\left(n-\left|A\right|-1\right)!\,\left|A\right|!}{n!}\,\left[\mu \,\left(A\cup \left(i\right)\right)-\mu \,\left(A\right)\right]$$

The Shapley value of *i* can be regarded as an average value of marginal contribution μ(*A*∪(*i*))−μ(*A*) of criterion *i* to a subset *A* not containing it. Another critical concept is the Shapley interaction index proposed by Murofushi and Soneda (1993), considering two criteria *i* and *j*, which is defined by


(3) $$I_{i\,j}=\sum_{A\subseteq N\,\left(i,j\right)}\frac{\left(n-\left|A\right|-2\right)!\,\left|A\right|!}{\left(n-1\right)!}[\mu \left(A\cup \left(i,j\right)\right)-\mu \left(A\cup \left(i\right)\right)-\mu \left(A\cup \left(j\right)\right)+\mu \left(A\right)]$$

The Shapley interaction index *I*_*ij*_ can be explained as an average value of the additional value by putting *i* and *j* together with all combinations being considered. When *I*_*ij*_ is positive (or negative), then the interaction relationship between criteria *i* and *j* is said to be complementary (or redundant). Generally speaking, the Shapley value and the Shapley interaction index well explain the overall importance of a criterion and the interaction relationship among criteria.

In dealing with an MCDM problem, one could be curious about identifying the overall importance of every criterion and their mutual interactions. Usually, we address this identification issue by using learning data, its main idea is to minimize the residual error between the data and the model, which can be expressed as the following formula


(4) $$E^{2}\,\left(\mu \right)=\sum_{k=1}^{n}{\left(y_{k}-C_{\mu }\,\left(x_{k}\right)\right)}^{2}$$

where *x* denotes an input vector for the evaluation value of different criteria, *y* denotes the output value, and *C*_μ_ denotes the Choquet integral concerning the fuzzy measure μ.

### The Heuristic Least Mean Square Method

When the Choquet integral is used, Equation 4 can be solved by quadratic programming. Let *u* = [μ_1_,μ_2_,…,μ_*n*_,μ_1,2_,μ_1,3_,…μ_1,*n*_,…μ_*n*−1,*n*_,μ_1,2,3_,……μ_2,3,…,*n*_]*^T^* be a (2^*n*^−2)-dimensional vector involving fuzzy measures of all the subsets, except for μ(ϕ) = 0 and μ(*N*) = 1. Accordingly, Equation 4 can be converted to a quadratic program form (Grabisch et al., 2013)


$$\mathrm{minimize}\,\frac{1}{2}\,u^{T}\,D\,u+c^{T}\,u$$

under⁢the⁢constraint⁢A⁢u+b≥0

where *D* is a(2^*n*^−2)-dimensional square matrix, *c* is a (2^*n*^−2)-dimensional vector, *A* is a *n*(2*^n^*^−1^−1)×(2^*n*^−2)-dimensional matrix and *b* is a *n*(2*^n^*^−1^−1)-dimensional vector. This identification method can be solved using the Lemke algorithm and there is no unique solution in general.

The least-squares capacity identification mentioned above provides an optimal solution, but in some cases (large *n* or few data), the issue becomes ill-conditioned thus bad results occur. In addition, the obtained optimal solution does not always meet the requirements of the decision-maker if an extreme value (near 0 and 1) occurs or is away from the equilibrium point μ(*A*) = 1/|*A*| for all *A*⊆*N*. Therefore, instead of the minimization of explicit criteria, several iterative algorithms were introduced based on intuitive considerations, one of these was a suboptimal algorithm introduced by Grabisch (1995), named the heuristic least mean squares (HLMS), which is based on the concept of equilibrium point and the gradient algorithm.

There are two fundamental steps in the HLMS identification method. Step one is done several times for all learning data to maintain the monotonicity property. Step two aims to get a lattice as homogeneous as possible by applying the language of pattern recognition, and expect less overtraining or better generalization ability.

### The Sample

The sample used in the present paper comes from the fifth wave of China Family Panel Studies (CFPS) launched by Peking University in 2018, which covers 34 provinces/municipalities/autonomous regions, representing most of the Chinese population (Institute of Social Science Survey and Peking University, 2015). CFPS is a practically comprehensive, nationwide, longitudinal general social survey project, its purpose is to document social phenomena changes in population, economy, education, and health in contemporary China (Xie and Hu, 2014).

The fifth wave of the full sample follow-up survey in 2018 contains 997 variables and 32,669 samples, among them, we choose those who are employed. After eliminating missing data and outliers, a total of 5,829 samples are obtained eventually. The mean age of this sample is 40.48 years old, 30.69% of whom were born in the 1980s and 26.68% of whom were born after the 1990s. 56.48% of the sample are male and 43.52% are female. And 72.24% are married people. The proportion of people with higher education is 33.61%. On the subject of geographical distribution, samples from Eastern China and Southwest China account for about 20% each, and the sample size of Central China and Northwest China accounts for about 17.4% and 14.96% respectively. Most of the employees in the sample (about 20.9%) are from manufacturing-related companies, this distribution fits well with China’s status as a manufacturing powerhouse, and their average annual income is 50,559 Yuan.

## Results

### Preliminary Design

Herzberg introduced the two-factor theory of job satisfaction in 1959, which divided the factors affecting job satisfaction into two categories: hygiene factors and motivation factors (Herzberg, 2017). Hygiene factors cannot generate satisfaction but can facilitate dissatisfaction with no dissatisfaction or short-term motivation (Raziq and Maulabakhsh, 2015), like salary, job security, working environment, company policy, and administration. Motivation factors can promote positive job attitudes and change no dissatisfaction into satisfaction, factors like recognition, responsibility, achievement, and work itself. Based on the two-factor theory, we select job income satisfaction, job safety satisfaction, working environment satisfaction, working time satisfaction, and job promotion satisfaction from the existing indicators of CFPS questionnaires to determine the overall job satisfaction, all measured on a 5-point scale with 5 representing the most positive response.

According to Shin and Johnson (1978), life satisfaction depends on the comparison of a person’s environment with the criteria considered appropriate. Likewise, Diener et al. (1985) put forward that although health and vitality may be desirable, particular individuals may have different values on them. That is to say, people’s satisfaction with the status quo is based on comparisons with the standards each person sets for themselves, it focuses on a person’s judgment rather than some criteria that researchers consider important. For that reason, the selected sub-criteria of life satisfaction include “How satisfied are you with your life?” “What is your relative income level in your local area?” “What is your social status in your local area?” “Do you think you are popular?” and “How confident are you about your future?”

Regarding positive and negative emotions, we select all eight indicators in questionnaires. These eight indicators give some descriptions of people’s mental statuses in the past week, two for positive emotions, and the other six for negative emotions. See Table 1 for details.

**TABLE 1:** The preliminary criteria system of employee well-being based on CFPS.

Criteria	Sub-criteria
Job satisfaction (A)	Job income satisfaction (A1)
	Job safety satisfaction (A2)
	Working environment satisfaction (A3)
	Working time satisfaction (A4)
	Job promotion satisfaction (A5)
Life satisfaction (B)	How satisfied are you with your life? (B1)
	What is your relative income level in your local area? (B2)
	What is your social status in your local area? (B3)
	Do you think you are popular? (B4)
	How confident are you about your future? (B5)
Positive emotions (C)	I feel happy. (C1)
	I have a happy life. (C2)
Negative emotions (D)	I am in a low spirit. (D1)
	I find it difficult to do anything. (D2)
	I cannot sleep well. (D3)
	I feel lonely. (D4)
	I feel sad. (D5)
	I feel that I cannot continue with my life. (D6)

Given the above, we finally get 18 sub-criteria based on CFPS in the preliminary design part, the item number of each indicator is given in parentheses. Among them, there are five sub-criteria for job satisfaction (*M* = 3.677, SD = 0.728, α = 0.805) and life satisfaction (*M* = 3.636, SD = 0.599, α = 0.709) respectively, two sub-criteria representing positive emotions (*M* = 1.515, SD = 0.368, α = 0.707), and the remaining six sub-criteria for negative emotions (*M* = 5.195, SD = 0.716, α = 0.779).

Table 2 shows the mean, standard deviation, and correlation coefficients of the sub-criteria. It can be seen from the table that the sub-criteria of each criteria group are significantly correlated. According to confirmatory factor analysis, the overall KMO measure is 0.822 (*p* < 0.001), indicating a good data structure and correlation. Table 3 illustrates the rotated component matrix of each sub-criteria. Component 1 explains 22.61% of the variation, mainly reflecting the sub-criteria of negative emotions, component 2 mainly reflects the sub-criteria of job satisfaction, component 3 can be interpreted as a sub-criteria of life satisfaction, and component 4 can be characterized by the sub-criteria of positive emotions. The results of the confirmatory factor analysis are consistent with our preliminary design.

**TABLE 2:** Mean, standard deviation (S.D.) and correlation coefficients of sub-criteria.

	Mean	S.D.	A1	A2	A3	A4	A5	B1	B2	B3	B4	B5	C1	C2	D1	D2	D3	D4	D5
A1	0.7	0.2																	
A2	0.784	0.179	0.417**																
A3	0.757	0.188	0.426**	0.570**															
A4	0.744	0.199	0.382**	0.445**	0.483**														
A5	0.688	0.208	0.486**	0.396**	0.467**	0.455**													
B1	0.809	0.171	0.250**	0.150**	0.207**	0.213**	0.257**												
B2	0.61	0.189	0.334**	0.149**	0.185**	0.177**	0.262**	0.347**											
B3	0.621	0.197	0.192**	0.128**	0.156**	0.150**	0.235**	0.324**	0.577**										
B4	0.744	0.162	0.123**	0.106**	0.116**	0.114**	0.181**	0.233**	0.218**	0.275**									
B5	0.853	0.157	0.191**	0.136**	0.166**	0.143**	0.235**	0.470**	0.281**	0.282**	0.269**								
C1	0.74	0.213	0.093**	0.088**	0.099**	0.121**	0.095**	0.158**	0.080**	0.092**	0.110**	0.142**							
C2	0.775	0.206	0.110**	0.092**	0.105**	0.126**	0.114**	0.215**	0.091**	0.117**	0.116**	0.196**	0.547**						
D1	0.82	0.178	0.096**	0.061**	0.056**	0.065**	0.090**	0.205**	0.079**	0.096**	0.116**	0.152**	0.163**	0.186**					
D2	0.839	0.189	0.040**	0.059**	0.037**	0.064**	0.027*	0.103**	0.029*	0.029*	0.070**	0.124**	0.144**	0.142**	0.485**				
D3	0.815	0.217	0.092**	0.053**	0.066**	0.088**	0.070**	0.118**	0.067**	0.057**	0.064**	0.104**	0.140**	0.135**	0.330**	0.345**			
D4	0.886	0.173	0.048**	0.044**	0.057**	0.048**	0.042**	0.174**	0.055**	0.063**	0.102**	0.129**	0.144**	0.202**	0.399**	0.360**	0.314**		
D5	0.875	0.162	0.084**	0.060**	0.062**	0.071**	0.047**	0.172**	0.079**	0.075**	0.114**	0.149**	0.173**	0.210**	0.440**	0.390**	0.292**	0.511**	
D6	0.959	0.118	0.026*	0.044**	0.026*	0.024	−0	0.100**	−0	0.001	0.057**	0.114**	0.113**	0.145**	0.306**	0.329**	0.246**	0.367**	0.434**

**p < 0.01; *p < 0.05, levels of significance.

**TABLE 3:** The rotated component matrix of each sub-criteria.

Sub-criteria	Component			
	
	1	2	3	4
D5	0.740			
D4	0.710			
D2	0.710			
D1	0.708			
D6	0.647			
D3	0.574			
A3		0.798		
A2		0.776		
A4		0.732		
A5		0.695		
A1		0.671		
B3			0.768	
B2			0.750	
B1			0.635	
B5			0.612	
B4			0.516	
C1				0.856
C2				0.844

### Measurement Model

In this part, we plan to apply multiple linear regression analysis and fuzzy analysis to obtain the measuring model of employee well-being and Shapley values of each indicator set. The appraisal of SWB is scored by the question “Are you happy?” measured from 0 to 10, and “0” is the lowest score while “10” is the highest. The average SWB score of the 5,829 samples is 8.1, with a score greater than 6 accounting for almost 95.3% of the sample, which means that most employees in this sample experience a high level of happiness.

Table 4 shows the results of simple multiple linear regression. Model 1 regresses all the 18 sub-criteria, the adjusted R-squared is 0.4445, but the coefficients of sub-criteria A2, A5, B2, and D3 are not significant at all. In model 2, the coefficients of sub-criteria D2 and D5 are relatively low (*p* < 0.05). After eliminating the sub-criteria “I find it difficult to do anything.” and “I feel sad.” we can have model 3 with the final 12 sub-criteria, its adjusted R-squared is 0.4421 and all the coefficients of the sub-criteria are significant at the level of *p* < 0.001. The final regression result can be expressed in Equation 5, where *n* = 5,829 and *R*^2^ = 0.4432. Above all, we can get the employee well-being evaluation criteria system, see Table 5.


(5) $$E\,W\,B=0.0452\,A_{1}+0.0492\,A_{3}+0.0491\,A_{4}+0.2326\,B_{1}$$

$$+0.0457\,B_{3}+0.2242\,B_{4}+0.1411\,B_{5}+0.0352\,C_{1}$$

$$+0.0740\,C_{2}+0.0730\,D_{1}+0.0743\,D_{4}+0.1065\,D_{6}$$

-0.1087

**TABLE 4:** Multiple linear regression results for preliminary sub-criteria on SWB.

Sub-criteria	Model 1	Model 2	Model 3
A1	0.0353***	0.0449***	0.0452***
	(3.62)	(4.99)	(5.01)
A2	0.0203		
	(1.85)		
A3	0.0384***	0.0496***	0.0492***
	(3.53)	(5.05)	(5.00)
A4	0.0405***	0.0476***	0.0491***
	(4.24)	(5.21)	(5.36)
A5	0.0146		
	(1.55)		
B1	0.2323***	0.2332***	0.2326***
	(21.16)	(21.43)	(21.35)
B2	0.0079		
	(0.75)		
B3	0.0411***	0.0461***	0.0457***
	(4.16)	(5.37)	(5.32)
B4	0.2219***	0.2233***	0.2242***
	(21.77)	(21.96)	(22.01)
B5	0.1364***	0.1387***	0.1411***
	(11.81)	(12.06)	(12.26)
C1	0.0322***	0.0330***	0.0352***
	(3.71)	(3.80)	(4.05)
C2	0.0729***	0.0728***	0.0740***
	(7.94)	(7.94)	(8.06)
D1	0.0511***	0.0539***	0.0730***
	(4.74)	(5.04)	(7.45)
D2	0.0244*	0.0276**	
	(2.47)	(2.82)	
D3	0.0147		
	(1.86)		
D4	0.0555***	0.0577***	0.0743***
	(5.07)	(5.31)	(7.28)
D5	0.0406**	0.0411**	
	(3.33)	(3.37)	
D6	0.0848***	0.0864***	0.1065***
	(5.63)	(5.75)	(7.41)
Constant	−0.1186***	−0.1184***	−0.1087***
	(−7.11)	(−6.84)	(−6.59)
Adjusted R-squared	0.4445	0.4440	0.4421

T-statistics in parenthesis; *p < 0.05; **p < 0.01; ***p < 0.001.

**TABLE 5:** The employee well-being evaluation criteria system based on CFPS.

Criteria	Sub-criteria
Job satisfaction (A)	Job income satisfaction (A1)
	Working environment satisfaction (A3)
	Working time satisfaction (A4)
Life satisfaction (B)	How satisfied are you with your life? (B1)
	What is your social status in your local area? (B3)
	Do you think you are popular? (B4)
	How confident are you about your future? (B5)
Positive emotions (C)	I feel happy. (C1)
	I have a happy life. (C2)
Negative emotions (D)	I am in a low spirit. (D1)
	I feel lonely. (D4)
	I feel that I cannot continue with my life. (D6)

To identify fuzzy measures for each criterion set in Table 5, we introduce the HLMS algorithm from the Kappalab toolbox. Kappalab stands for “laboratory for capacities,” which is a package for the GNU R statistical system. The mean square error between the provided SWB scores and those returned by the obtained model is 0.005, the Shapley value and interaction indexes of sub-criteria are presented in Table 6. The second column in Table 6 is the Shapley values for each sub-criteria, which represent the weight of a given criterion in the model. A1 has the lowest weight of 0.064 and is relatively unimportant among all the 12 indicators, which can explain 6.2% of employee well-being. In contrast, B4 has the largest weight of 0.094, explaining 9.4% of employee well-being. The Shapley interaction indexes in Table 6 model the concept of interaction between sub-criteria, there are two types of interaction relationships: positive (complementary) and negative (redundant). For instance, the Shapley value of A1 is 0.064, and the Shapley value of A3 is 0.068, the value of the Shapley interaction index (A1, A3) is −0.00302, which is a negative value, indicating that the relationship between the two is redundant, because both job income satisfaction and working environment satisfaction can be classified as hygiene factors. It should be noted that the relationships between positive emotions and negative emotions are not always complementary, there is some redundancy between them, which is consistent with the argument that positive and negative emotions are not opposite, but moderately negatively correlated (Diener et al., 2018). Moreover, the relationships between the indicators related to positive and negative emotions and those related to life satisfaction and job satisfaction are mostly redundant, which indicates that emotions and satisfaction should not be confused, it is not appropriate to assess only life satisfaction or job satisfaction to comprehensively evaluate individual SWB.

**TABLE 6:** The Shapley value and interaction indexes of sub-criteria.

	Shapley value	A1	A3	A4	B1	B3	B4	B5	C1	C2	D1	D4
A1	0.06407											
A3	0.06815	–0.003										
A4	0.08087	0.01023	–0.0118									
B1	0.08501	0.00758	0.00851	0.00217								
B3	0.08607	0.01089	–0.0034	–0.001	–0.0039							
B4	0.09357	0.00086	0.01009	0.00543	–0.0127	–0.0047						
B5	0.08936	0.00369	0.00098	–0.0021	0.00208	0.00233	–0.0048					
C1	0.08464	–0.0032	–0.0018	–0.0026	–0.0011	0.00812	0.00272	–0.0106				
C2	0.0855	–0.0017	0.0059	–0.003	–0.0026	–0.0036	–0.0048	0.01335	0.00097			
D1	0.08697	–0.0082	0.00152	0.00349	–0.0088	–0.0055	0.00838	–0.0019	0.00625	–0.0007		
D4	0.08656	–0.0111	–0.006	0.0036	0.00133	–0.008	0.01459	–0.004	–0.0029	–0.0081	0.00836	
D6	0.08924	–0.006	–0.0009	–0.0044	0.00192	0.00169	–0.0139	0.00088	0.00422	0.00298	–0.0004	0.01218

In Table 7, the average coefficient value comes from multiple linear regression analysis, and the average Shapley value comes from the fuzzy analysis. The results of both analyses show that job satisfaction has the lowest weight and life satisfaction has the highest weight. Bryson et al. (2017) mentioned that some psychologists have criticized the study for focusing too much on job satisfaction and not enough on other aspects of SWB. The research results of this paper also verify that job satisfaction cannot fully explain employee well-being, and more attention should be paid to other aspects of employees, such as life satisfaction because Table 7 shows that life satisfaction is significantly more important than other dimensions. In addition, negative emotions also have a significant impact on employee well-being, indicating that a certain degree of negative emotions may also have a positive impact on employees, because people in a negative emotional state tend to work harder, more systematically, and process more fragmented information (Edwards and Weary, 1993), and they tend to make more accurate, impartial, and realistic judgments (Sinclair, 1988). It is said happiness seemed to consist of frequent positive emotions and infrequent negative emotions (Diener et al., 2009b), therefore, we believe that the decrease in the frequency of negative emotions is more conducive to the improvement of employee well-being than the increase in the frequency of positive emotions.

**TABLE 7:** The average Shapley value and coefficient of four criteria.

Criteria	Job satisfaction (A)	Life satisfaction (B)	Positive emotions (C)	Negative emotions (D)
The average coefficient	0.048	0.161	0.055	0.085
The average Shapley value	0.071	0.089	0.085	0.088

### Fitting Analysis

**FIGURE 1:**
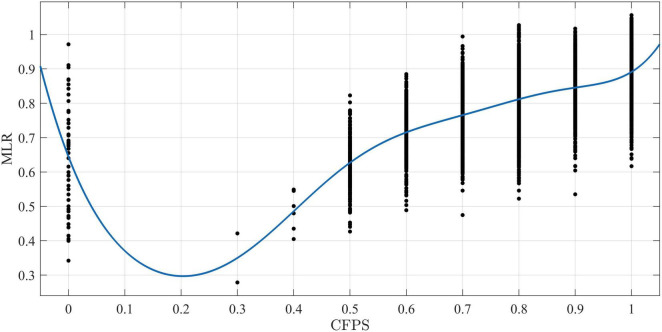
The figure fits the Fourier curves of CFPS data and MLR data. The software reports that the sum of squares for error (SSE) is 32.55, the R-square coefficient is 0.448, and the root mean square error (RMSE) is 0.075. The analysis of the three fitting data shows that the fitting effect of MLR is not good, because SSE is too large and the R-square coefficient is less than 0.5.

**FIGURE 2:**
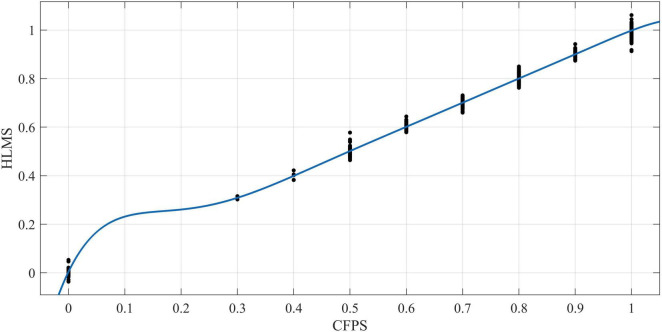
The figure fits the Fourier curves of CFPS data and HLMS data. The software reports that the SSE is 0.345, the R-square coefficient is 0.998, and the RMSE is 0.008. These three indicators all reveal that the HLMS algorithm has a good fitting degree with the truth curve, and the curve trend further illustrates the accuracy and effectiveness of the HLMS algorithm.

There is a total of 5,829 samples, which are modeled by multiple linear regression (referred to as MLR) and the HLMS method (referred to as HLMS). Fast Fourier transform (FFT) is a fast algorithm of discrete Fourier transform, which is obtained by improving the algorithm of discrete Fourier transform according to the odd, even, virtual and real properties of discrete Fourier transform (Brigham, 1988). FFT has been widely used in signal processing and curve-fitting due to its obvious advantages of small computation. Figures 1, 2 are obtained by the FFT fitting method in MATLAB software.

## Discussion

Based on the sample data of CFPS, this study constructs a multi-criteria decision model of employee well-being, which contains 4 criteria and 12 sub-criteria. The relationship among the criteria is discussed by using multiple linear regression analysis and fuzzy analysis. The main conclusions are as follows:

(1) According to the literature review, this study proposes that employee well-being can be divided into four components: the presence of positive emotions, the absence of negative emotions, life satisfaction, and job satisfaction. These four dimensions do not exist independently, they are dependent and related to each other. Therefore, the measurement of employee well-being is complex and fuzzy, which cannot be precisely defined and expressed with a clear value.
(2) It is not appropriate to use job satisfaction as a direct substitute for measuring employee well-being. According to the multiple linear regression analysis and fuzzy analysis in this paper, the weight of job satisfaction is the lowest among the four dimensions, which indicates that job satisfaction cannot be completely equal to the overall well-being of employees. In addition, the analysis also shows that the weight of life satisfaction is the largest among the four dimensions, which implies that employee well-being is also reflected in their overall satisfaction with life to a large extent.
(3) Employee well-being requires comprehensive consideration of emotion-related indicators and satisfaction-related indicators. The Shapley interaction index shows that the relationship between positive and negative emotions is not always complementary, there is some redundancy between them, and they are moderately negatively correlated. In addition, fuzzy analysis proves that the relationship between emotion-related indicators and satisfaction-related indicators is mostly redundant, indicating that emotion and satisfaction should not be confused, and it is biased to use life satisfaction or job satisfaction to comprehensively evaluate individual well-being.

### Practical Implications

There are several practical implications: Firstly, the multi-criteria model of employee well-being developed in this study promotes the understanding of employee well-being in the field of positive psychology, especially the analysis of the multi-dimensional theoretical model of employee well-being from the perspective of fuzzy measure, which has important theoretical value for the study of employee well-being. Secondly, organizations should not only pay attention to employees’ job satisfaction but also pay attention to their life satisfaction. Organizations should reasonably arrange employees’ work tasks and promote work-family balance in order to effectively improve employee well-being. Thirdly, organizations should strengthen the emotional management of employees, maintain and stimulate employees’ positive emotions at work, and help vent their negative emotions, so that employees can have a better emotional experience at work. Finally, organizations can apply the multi-criteria model in this paper to understand the current situation of employee well-being, so as to formulate appropriate human resource management strategies and achieve the purpose of prompting employees’ overall well-being and enhancing organizational performance.

### Limitations and Future Directions

This study has the following limitations and corresponding future research directions: Firstly, the research conclusions may not be universal, because the data in this paper are from China. A country’s system, education, technology, and culture will lead to differences in employees’ behaviors and psychological states. Therefore, in order to better understand employee well-being, future research can focus on employee well-being in different countries to reveal differences. Secondly, employees’ judgment of their own emotions and satisfaction is dynamic. However, this paper only analyzes the cross-sectional data of 2018, and the dynamic panel data may help to obtain more stable research conclusions. Subsequent studies can use dynamic panel data to build a measuring model of employee well-being.

## Data Availability Statement

Publicly available datasets were analyzed in this study. The datasets analyzed for this study can be found in the public social science survey panel data from Peking University named China Family Panel Studies (https://opendata.pku.edu.cn/dataverse/CFPS).

## Author Contributions

JD conceived of the initial idea, designed the study, collected and analyzed the data, and drafted the manuscript. JD and SY revised and proofread the manuscript. Both authors contributed to the article and approved the submitted version.

## Conflict of Interest

The authors declare that the research was conducted in the absence of any commercial or financial relationships that could be construed as a potential conflict of interest.

## Publisher’s Note

All claims expressed in this article are solely those of the authors and do not necessarily represent those of their affiliated organizations, or those of the publisher, the editors and the reviewers. Any product that may be evaluated in this article, or claim that may be made by its manufacturer, is not guaranteed or endorsed by the publisher.
